# A novel and generalizable organotypic slice platform to evaluate stem cell potential for targeting pediatric brain tumors

**DOI:** 10.1186/1475-2867-8-9

**Published:** 2008-05-22

**Authors:** Shengwen Calvin Li, William Gunter Loudon

**Affiliations:** 1 Center for Neuroscience and Stem Cell Research, Neuroscience Institute, Children's Hospital of Orange County Research Institute, 455 S. Main Street, Orange, CA 92868, USA; 2 Department of Neurology, University of California Irvine, Orange, CA 92862-4280, USA; 3 Department of Neurological Surgery, University of California Irvine, Orange, CA 92868-3298, USA; 4 Department of Cancer Biology, Kimmel Cancer Center, Thomas Jefferson University, Philadelphia, PA 19107, USA; 5 Department of Biological Science, California State University, Fullerton, CA 92834, USA

## Abstract

Brain tumors are now the leading cause of cancer-related deaths in children under age 15. Malignant gliomas are, for all practical purposes, incurable and new therapeutic approaches are desperately needed. One emerging strategy is to use the tumor tracking capacity inherent in many stem cell populations to deliver therapeutic agents to the brain cancer cells. Current limitations of the stem cell therapy strategy include that stem cells are treated as a single entity and lack of uniform technology is adopted for selection of clinically relevant sub-populations of stem cells. Specifically, therapeutic success relies on the selection of a clinically competent stem cell population based on their capacity of targeting brain tumors. A novel and generalizable organotypic slice platform to evaluate stem cell potential for targeting pediatric brain tumors is proposed to fill the gap in the current work flow of stem cell-based therapy. The organotypic slice platform has advantages of being mimic *in vivo *model, easier to manipulate to optimize parameters than *in vivo *models such as rodents and primates. This model serves as a framework to address the discrepancy between anticipated *in vivo *results and actual *in vivo *results, a critical barrier to timely progress in the field of the use of stem cells for the treatment of neurological disorders.

## Introduction: current challenges in treatment of pediatric brain tumors

Over 1.4 million people in the United States were diagnosed with cancer in 2007 and the national cost of the disease was over $206 billion in 2006, accounting one-thirds of healthcare dollars (total: $686 billion) spent in the U.S. [1,2]. An estimated 18,820 new cases of brain cancer was diagnosed in the United States of America in 2006, and more than 12,000 would die from the disease (data from the National Cancer Institute of the United States of America). Our current forms of therapy for these diseases are brain surgery followed by administration of toxic drugs and exposure to radiation, which lead that the patients face challenges due to both the effects of treatment and potential neurological dysfunction. Overall the cost of care per patient was $67,887 with accrued mean monthly health care costs that were 20 times higher than demographically similar individuals without cancer ($6364 vs. $277)[3].

Primary malignant tumors such as high grade gliomas diffusely migrate into the brain early in the disease course, disseminating tumor microsatellites to distant regions of the central nervous system [4]. These tentacles of tumor exist interspersed between normal functional tissues. Complete surgical resection of many malignant brain tumors is not practical by virtue of their anatomical location and the relationship of this diffuse disease relative to eloquent functional tissue. Adjuvant therapies including chemotherapy and radiation therapy are often used in conjunction with surgery for many types of cancer to attempt eradication of the residual tumor [5]. In malignant brain tumors, however, combined surgical and adjuvant therapies frequently prove insufficient to eliminate neoplastic disease as a result of unique characteristics of CNS anatomy and function as well as and practical limitations concerning biological characteristics of the tumor [6]. Therefore, despite gross total surgical resection, chemotherapy and radiation therapy, neoplastic cells persist and inevitably give rise to recurrent tumor. The majority of children with malignant glioma die and survivors are usually left with lifelong neurological and cognitive disabilities due to the cumulative result of pre-treatment damage arising from the growing tumor, and the deleterious effects of surgery and adjuvant therapies [7-13]. It is clear that a new medical approach to brain cancers is needed. Stem cells may provide the basis for a new approach.

## Emerging stem cell therapy of brain tumors

The lack of efficacy for conventional treatments of malignant brain tumors can be readily appreciated by the grave prognosis of for malignant gliomas, brainstem gliomas. In contrast to pediatric hematological malignancies, meaningful improvements in survival statistics for patients with malignant brain tumors have not been realized in over thirty years of clinical research [14]. New strategies which circumvent the limitations of conventional brain tumor treatments must be conceived, tested and applied to this devastating disease. One such emerging strategy is to use the tumor-tracking capacity apparently inherent in many stem cell (As defined by their capacity of self-renewal and multipotency) populations tested to identify, track and potentially effect therapeutic modulation of the brain tumor microenvironment [15], lessening the reliance on the current treatment methods [16-18]. Potential stem cell populations for clinical application include hematopoietic stem cells, human brain-derived neural stem cells (NSC), bone marrow-derived mesenchymal stem cells (MSC), embryonic stem cell-derived human NSC (eNSC), umbilical cord blood derived stem cells, and human amniotic fluid stem cell. Several intrinsic issues in embryonic stem cell therapy include: 1) wide ranging ethical consideration; 2) the availability of these cells for clinical use are limited; and 3) immunosuppression is required for successful engraftment, which may compromise overall patient conditions.

The emerging evidence shows a promising result for use of stem cells for the treatment of brain cancers. Aboody and colleagues were the first to demonstrate that neural stem cells (NSC), when implanted into experimental intracranial gliomas *in vivo *in adult rodents, target themselves throughout the tumor bed [19]. The implanted NSC migrates through normal tissue targeting the distantly implanted tumor cells. When implanted outside the CNS intravascularly, NSC can also target an intracranial tumor. As such, the intracranial and intravenous administration of inherently migratory NSC may be used as a delivery vehicle for targeting therapeutic agents to modulate brain tumors. It was shown that the endogenous NSC were specifically activated and mobilized into an intracranial tumor while such migratory activity was not observed in the setting of other nonneoplastic lesions [20,21]. More importantly, inherent anti-tumor properties within NSC themselves was reported: Exogenously administered unmodified NSC inhibited glioma proliferation *in vivo *and conditioned medium from NSC suppressed the proliferation of tumor GL261 cells *in vitro *[22].

Marrow stem cells (MSC) are good candidates for such a cell-based therapy in human. The main advantages of using MSC for replacement therapy are that MSC: a) are the readily accessible and large quantities can be harvested using patient's own stem cells (autologously) for future clinical applications, b) can be genetically modified with therapeutic genes with high efficiency without loss of stem cell capacity, c) support sustained expression for specific therapeutic proteins, d) support auto-transplantation, not requiring immunosuppression; and e) are generally not subject to ethical concerning like those associated with the use of human embryonic stem cells or fetal neuronal stem cells. In fact, MSC shows an extensive tropism to gliomas, which actively attract MSC by secreting a multitude of angiogenic and chemotactic cytokines IL-8, TGF-β1, NT-3, SDF-1, and VEGF [23-25]. Studeny's group pioneered to demonstrate that MSC can be integrated into the tumor architecture, which inhibit tumor growth *in vivo *by local production of an anti-tumor molecule interferon (IFN-β) transduced in the MSC [26]. Such an MSC-assisted targeting delivery of IFN-β to the tumor bed may be advantageous because the excessive toxicity associated with systemic administration at effective doses limits its use as a clinically viable therapeutic modality. Strikingly, this prediction was confirmed by an observation that significantly extending length of survival of mice harboring human gliomas is achieved with transplanted MSC carrying IFN-β gene but not with intravenous injection of IFN-β. It appears that MSC can track down not only the main tumor mass but tumor satellites infiltrated deep into normal neural tissue [27].

## Clinical trials using adjuvant stem cells for treatment of pediatric brain tumors

Current clinical application of stem cells is considered as part of combination treatment of brain tumors of using surgical, chemotherapy and radiation therapy to rejuvenate blood and immune systems. Only limited complete data sets are available currently even though autologous hematopoietic stem cell transplantation (AHSCT) has been advocated as a form of salvage therapy for children with high-risk or relapsed brain tumors for decades [28].

Worldwide there are 38 registered stem cell-based clinical trials for variety of pediatric brain and central nerve system (CNS) tumors; most of which are in Phases I/II (see the Additional file 1). Majority of these trials are for intravenous administration of autologous transplants using bone marrow and peripheral blood derived stem cells and only one study (NCT00005796) is conducted with *in vitro *treated peripheral blood stem cells, which are Fibronectin-assisted, retroviral-mediated modification of CD34+ PBSC carrying O^6^-methylguanine DNA methyltransferase. The rationale for using the stem cell is not to directly target the tumor but to replace immune cells that were destroyed by chemotherapy.

In the Schneider Children's Medical Center of Israel Rabin Medical Center (NCT00607984), patients with metastatic and relapsed brain tumors were treated with a high-dose chemotherapy (HDC) followed by autologous stem cell transplantation (ASCT) as the consolidation therapy. A large randomized study in children with high risk neuroblastoma showed that application of autologous stem cell transplant can lead to improved disease free and overall survival, effects that were further augmented by the administration of biological agents with specific activity against this tumor. Smaller non-controlled studies and case series have shown that ASCT is feasible in children with solid tumors or with tumors of the central nervous system.

A major limitation of many high-dose chemotherapy (HDC) protocols, experimental gene therapies, and biologic therapies is that the administered agents are unable to traverse the blood brain barrier (BBB) in order to reach the site of the tumor. Thiotepa, a highly myeloablative bifunctional alkylating agent, was considered a major breakthrough in the application of high dose chemotherapies in children with CNS tumors because it partitions equally across the BBB. Thiotepa remains a mainstay of HDC protocols for children with CNS tumors. As such, combining chemotherapy with peripheral stem cell transplantation may allow the doctor to give higher doses of chemotherapy drugs and kill more tumor cells. Similarly, TEMOZOLOMIDE (Temodor) is the American Food and Drug Administration (FDA) approved for grade III anaplastic astrocytoma based largely on low side effects profile and excellent CNS penetration. Preliminary studies suggest that stem cells can cross BBB to reach brain tumors targets. Alternatively, therapeutic stem cells population can be directly introduced via stereotactic injection into the CNS tumor tissue.

Cheuk and colleagues report the long-term effects of AHSCT treatment for 13 pediatric brain tumor patients for a period of 10 years (1996–2006) in Hong Kong, including medulloblastoma (n = 9), cerebral primitive neuroectodermal tumor (n = 1), ependymoma (n = 1), germ cell tumor (n = 1) and cerebellar rhabdoid (n = 1) with tumor residual (n = 1) or recurrence (n = 12). Prior to AHSCT, 8 patients (61.5%) achieved complete remission and 5 (38.5%) were in partial remission with conditioning employed thiotepa, etoposide and carboplatin. Adverse effects of mucositis and neutropenic fever in all patients, grade 4 hepatic toxicity in 4 patients and grade 4 renal toxicity in 1 patient were observed associated with the chemotherapy. The results with AHSCT in the study include: 1) The 5-year event-free survival was 53.9%; 2) Five patients died of disease recurrence or progression 8–21 months after transplant with a median disease-free period of 8 months post-transplant; 3) One died of transplant-related complications in the early post-transplant period; 4) Seven survived for a median of 5.4 years (maximum follow-up of 9.8 years), with 6 having Lansky-Karnofsky performance score above 80; 5) All survivors had complete remission before transplant though 2 had leptomeningeal spread [28]. It appears that AHSCT can empower long-term survival in children with recurrent brain tumor, however; those patients with macroscopic residual tumors before the transplant could not be salvaged.

Nonetheless, controversial efficacies raised doubt about the clinical benefits of conventional stem cell therapy. The efficacy can be fluctuated by many factors including the quality of the stem cell (number of effective stem cell sub-population, the differentiation status, and the age of the stem cell), the occurrence of graft versus host reaction, overall survival, disease free survival and immune recovery [18]. Some of these efficacious variations may stem from the biological differences related to a cancer or treatment of interest. Others, however, may reflect the heterogeneity of patients across multiple sites, the inherent biological complexity and diversity of different cancer types, and even small differences in stem cell preparation, processing, handling, and analysis techniques used by multiple operators across multiple locations. As a consequence, data may be differed by site-, study-, population-, or sample-specific anomalies and, therefore, not be sufficiently robust for making a concrete conclusion. There is an urgent need for development of a uniform technical platform to clarify the ultimate clinical viability of stem cell therapy for brain tumors.

## Mechanism of action for stem cell therapy of brain tumors

Although stem cells show certain level of therapeutic benefits, little is known about mechanisms by which stem cells eradicate tumor cells by use of stem cell homing and migration toward the tumor. Stem cells may directly modulate the tumor microenvironment via their therapeutic effects of regenerative potential, neurotrophic and neuroprotective properties, and immune regulatory functions (e.g., inhibit the cellular inflammatory process in the tumor) [29,30]. Human MSC transcriptome analyses reveal that MSC transplanted at sites of nerve injury promote functional recovery by producing trophic factors that induce survival and regeneration of host neurons, including BDNF and β-NGF, various neurite-inducing factors, axon guidance and neural cell adhesion molecules [31].

Some examples of therapeutic strategies such as gene or drug delivery systems using stem cell homing show an anti-cancer efficacy, including EGFR antagonist, IL-2, IL-4 cytotoxin, interleukin-13 receptor-directed cytotoxin [32], BMP-1/-2, IFN-β, TNFα [33]. Intriguingly, efficacy was shown in three stem cell types tested, including brain-derived mouse neural stem cells (mNSC), brain-derived human NSC (hNSC), and embryonic stem cell-derived human NSC (eNSC) for treatment of neurometabolic disorders in the animal model [34]. No efficacy was shown in human fibroblasts, which stayed in the local injection site, suggesting that efficacious cell populations must possess the inherited physiologically-relevant properties such as the migrate capacity toward inflammation. Despite these exciting reports, many scientists have difficulties in duplicating the similar anti-tumor effects of NSC in their laboratories. It is possible that this property may vary with the source, method of preparation, differentiation status, and age of the stem cells. A screening platform is required to isolate and expand such a potent anti-cancer sub-population of the stem cell with the targeting capacity for clinical application.

## Current knowledge and unanswered questions in development of stem cells for targeting therapy of brain tumors using the conventional tissue culture and animal models

Despite exciting initial reports, clinical potency of stem cell therapy in animal brain tumor models has to date proven disappointing. Attempts to extrapolate current results to humans arrive at discouraging and impractical protocols. Indeed, some initial attempts to apply of human disease by embryonic stem cell therapy have proven dangerous to the subject, e.g., induced the formation of heterogeneous tumors and trigged inflammation. However, with optimization of clinically relevant parameters (enrichment and expansion of functional stem cell populations, improved recruitment and activation of clinically relevant stem cell populations, enhancement of targeted migration, and augmentation of therapeutic potency of targeted stem cells), realistic clinical protocols are anticipated.

**Figure 1:**
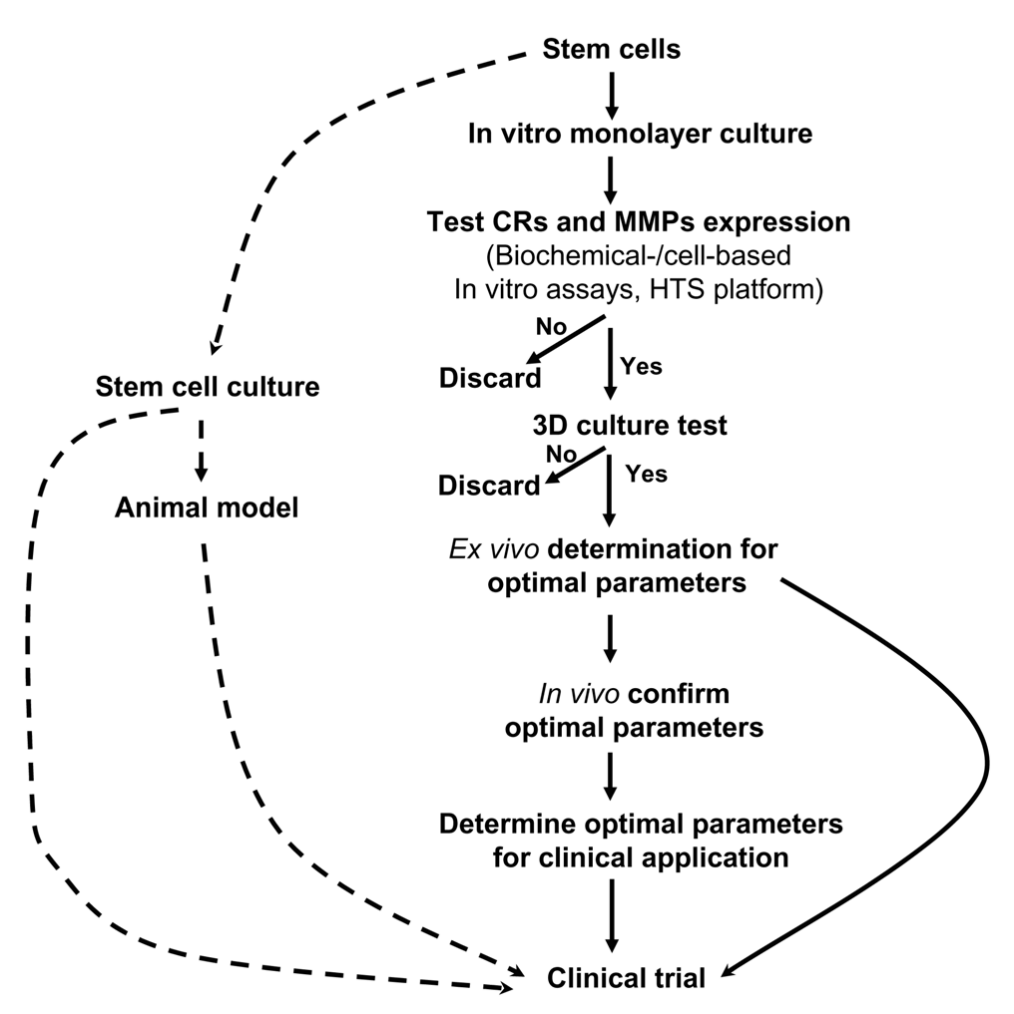
A workflow chart for development of stem cell sub-populations capable of targeting brain tumors for clinical application. CR: Chemokine receptor; HST: High throughput screening; MMPs: Metal metalloproteases; 3D: Three-dimensional culture. Dash line: Current workflow. Solid line: our proposed workflows.

Beside ethical and immunologic concerns associated with the use of fetal-derived tissue as a stem cell source, the problem is that lack of uniform platform in current stem cell research leads to an inconsistent efficacy. It takes a long time to select an effective tumor-tracking sub-population of the stem cell, even more challenge to optimize a new therapy with current workflow (Figure 1). The current technique includes determination of the source of working stem cells (MSC, NSC, eNSC), the selection of culture media, the state of stem cell cycle (undifferentiated vs. differentiated), the mode of culture (adhere vs. suspension), the form of growth (neurosphere vs. monolayer), substrate variations (Fibronectin, laminin, or Matrigel) and using different animal models to test each therapeutic variation, each possible way of using different stem cells (MSC, NSC, eNSC) to treat different brain cancers. An additional and very troublesome problem is that once stem cells are introduced into the animal, they become very difficult to track as they blend in with the animal tissue, since they are not an organ and they are very small. It becomes difficult to determine, exactly, where the cells are going and what the cells are doing. It is not uncommon that millions of stem cells are used for transplantation, however; only a tiny fraction of the stem cell is found in the target injury tissue [35]. As such, clinical progress is greatly hindered by current scheme of using *in vitro *assays and animal models, which result in inconsistent efficacies.

## An innovative organotypic slice platform for evaluation of stem cell potential to targeting brain tumor

**Figure 2:**
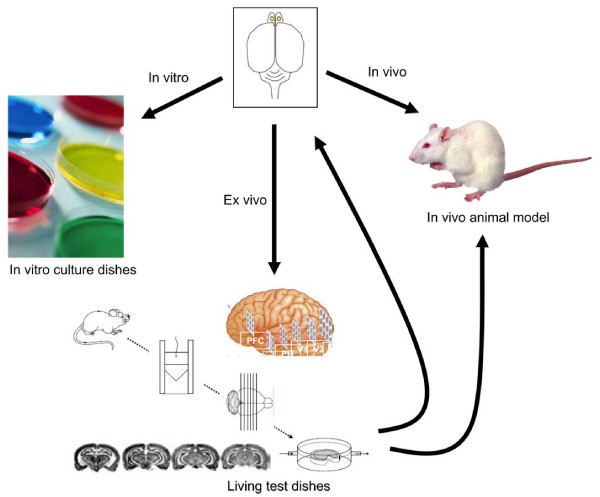
Schematic diagram for model systems of studying brain stem cells including in vitro, ex vivo and in vivo.

**Figure 3:**
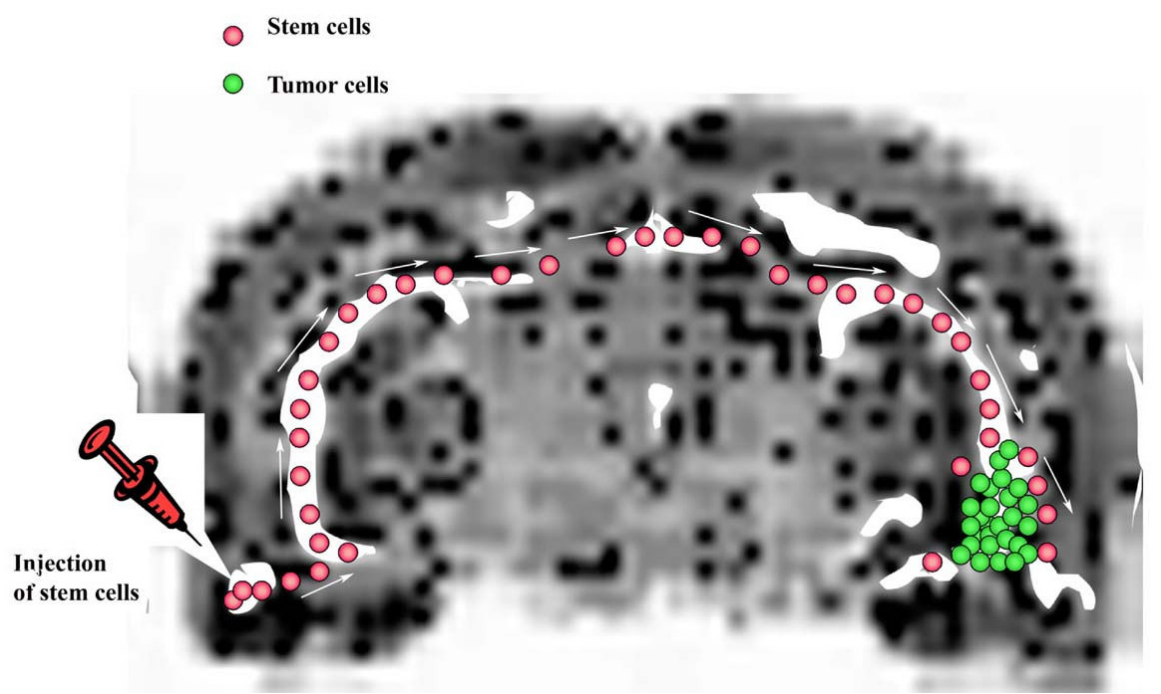
Stem cells showing tropism for malignant tumor cells implanted on rodent organotypic slice model. An organotypic slice is derived from a central nervous system tissue of an organism, indicating that the central nervous system tissue is sliced at a boundary such that an endogenous fiber tract of the central nervous system tissue is intact, through which the stem cell migrate toward the CNS tumor. Scheme is based on [15].

Targeting competency of stem cells is a quintessential stepping stone to successful stem cell therapy of brain tumors, however; only a fraction of cultured stem cells possess such a chief characteristics in stem cells prepared with conventional work flow (Figure 1) [36]. Our proposed workflow allows us to much more quickly identify and abandon techniques and stem cell populations that do not work and arrive at techniques and cell populations that do (Figure 1). We have established a platform technology, which is a much more efficient approach, namely "living test dish" (patent pending) or "brains in a dish" for selection of stem cell sub-populations capable of targeting brain tumors (Figure 2). The living test dish consists of organotypic slices that are micrometers thick of an animal organ, which are cultured under conditions in which the slice retains the cellular composition, morphology, and the physiological properties of the in situ source organ (Figure 3). The slice model presents a major advantage over using traditional *in vitro *cell culture methods and *in vivo *models (Table 1). By using living slices that viability of the slice can be maintained for many months, we can introduce stem cells into to these living "brains in a dish," determine cell populations in a real-time fashion. In this platform, we can serially manipulate and determine the parameters optimal for targeting brain tumors in a relatively short period of time before actually moving into experiments in the whole animal. This platform also allows us to use far fewer animals. Importantly, we believe that this organotypic slice-based approach lends itself to applications for a wide variety of other brain diseases and diseases of other organs of the body as well.

**Table 1:** Comparison of Three Test Systems: In vitro, ex vivo, and in vivo model.

	**In Vitro**	**Animal Model**	**Living Test Tube**
**Cost Effective**	+ + +	- - -	+ +
**Realistically Mimic In Vivo**	- - -	+ + +	+ + +
**Assay Multiple Time Points**	+ + +	-/+	+ + +
**3 Dimensional Integration**	- - -	+ + +	+ +
**Real-Time Monitor**	+ +	- -	+ + +
**High Throughput Screening**	+ + +	- - -	+ +
**Ethical (Animal)**	+ + +	- - -	+

Alternatively, an innovative organotypic slice system can be generated from a modified cellular component, a modified extracellular matrix component, a modified genetic component, or a combination thereof. For example, in the central nervous system, the extracellular matrix influences the interactions of neuronal cells and glial cells; and, it also regulates cell migration, cell survival, cell differentiation, axonal growth, and synapse formation. Such organotypic slice can be derived from a transgenic, mutant, null, gain-of-function, loss-of-function, knock-in, or knockout animals. The modified organotypic slice system can be used to dissect how implanted stem cells interact with resident cellular matrix and injured residential cells to predict how stem cells behave *in vivo*.

## Application of the organotypic slice platform for determination of targeting capacity of stem cells

**Figure 4:**
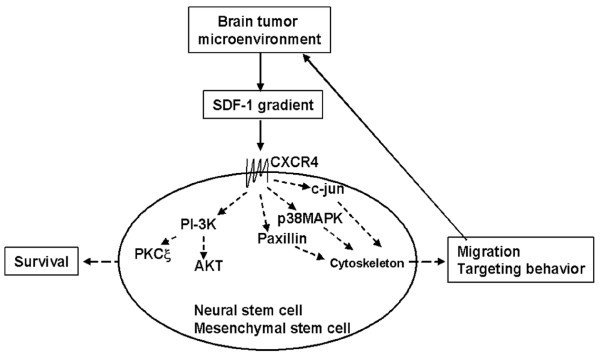
Model of SDF-1-mediated signal transduction for targeting of stem cells toward brain tumor. Brain tumors release SDF-1 gradient into the tumor microenvironment. Binding SDF-1 to its receptor expressed in stem cells triggers the signal transduction pathways that lead to cytoskeletons reorganization, which drives stem cell migration toward brain tumor microenvironment. CXCR-4: chemokine (C-X-C motif) receptor 4, a.k.a., SDF-1 receptor or CD184; SDF-1: stromal cell-derived factor-1 (chemokine), a.k.a., CXCL12 (see [37]).

For example, research efforts show that two properties of stem cell functional activity are critical: the ability of the stem cell to detect a target (homing) and the ability of the stem cell to track and migrate through the tissue to its target (matrix-remodeling) for their integration and differentiation to foci of intracranial glioma [36]. These two functions have been used interchangeably but they are distinct and equally important. Homing appears to be mediated to a large extent by the secretion of chemokines into the tumor microenvironment and the expression of chemokine receptors on the stem cells [37]. The essential driving force for stem cell migration toward brain tumors is the binding chemokine (e.g., SDF-1) to its chemokine receptor (e.g., CXCR4) that mediates the signal transduction (Figure 4). Matrix remodeling appears to be mediated, at least in part, by the secretion of matrix metalloproteinases by the stem cell [38]. Independent, but coordinated, regulation of these two functional behaviors of stem cells (therapeutic activation) must occur for these cells to be of any therapeutic competency. Our data argue for the requirement of "appropriate" extracellular matrix environment for optimal metalloproteinase expression (Manuscript in preparation).

Little is known about how we can effectively activate and program the stem cell migration for stem cell transplantation although it is known that regulators of migration include MMPs, elastases, c-kit, and stem cell factor which govern the expression of SDF-1 and CXCR-4 [36]. For example, quiescent stem cell populations minimally express chemokine receptors, which are not sufficient for any therapeutic success [39,40]. To compound the problem, different culture conditions affect the migratory parameters and migration pattern [41,42]. The further selection of a migratory stem cell sub-population necessitates a platform technology to identify and manipulate a "therapeutic migratory state" that addresses a potential window of opportunity when stem cells are the most effective to target brain tumors. A work flow for selection of both stem cell sub-population and their activation status is a quintessential stepping stone for any therapeutic success.

We postulate that an organotypic slice-based platform integrated with a workflow from an *in vitro *three dimensional extracellular milieu model (3D), an *ex vivo *organotypic brain slice model to the *in vivo *animal model ultimately can be used for selection of therapeutic migratory stem cell sub-population and their activation state (Figure 1). The platform of using organotypic slice can validate how temporal expression levels of chemokine receptors of the stem cells can be quantitatively correlated with the capacity of migration toward to brain tumor-produced signal ligand SDF-1. We can test experimentally this workflow paradigm. The foundation of this experimental platform is to establish a system mimic *in vivo*, first to maintain stem cells in a quiescent state, and then induce stem cells to produce targeting molecule cytokine receptors and matrix remodeling enzymes. We can show that stem cells remain quiescent without SDF-1 in three-dimensional culture. In the presence of SDF-1, stem cells are induced to produce MMP-9 and CXCR4. One critical issue is to determine if CXCR4 expression levels correlate with stem cell migration toward SDF-1 concentration gradients. Our data indicate that migration of neural stem cells is enhanced by an intermediate concentration of SDF-1 gradient but inhibited by higher concentrations, with no stimulation at low concentrations (Manuscript in preparation). These results may suggest that SDF-1 gradients may coordinate the stop and start signals for regulating stem cell migration. Another critical issue, assuming that receptor expression affects migration, asks whether CXCR4 expression in stem cells can be experimentally and even clinically manipulated. Our data indicates that CXCR4 mRNA and surface receptor expression increases with defined cultured medium and conditions (Manuscript in preparation).

**Figure 5:**
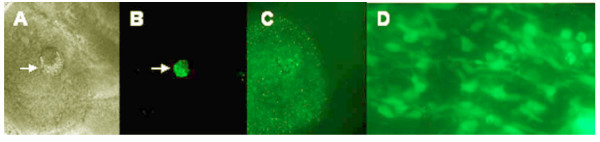
Rat brain organotypic slice can support stem cell survival and migration toward human tumor cells. A: Part of rat brain slice showing the implantation site (arrow) of GFP labeled neural stem cells (NSC) (phase contrast, 4×). B: Same field as (A) under fluorescence illumination showing the implanted cells (arrow). C: As early as 1 hour of implantation, NSC migrated out of the initial site toward a remotely implanted brain tumor site (not shown); D Live imaging of NSC 30 days after implantation indicating that NSC survived at least 30 days on the slice (40×).

The ultimate criterion is to test that expression scale of CXCR4 correlates the stem cell capacity of targeting brain tumors *in vivo*. Our hypothesis is that *ex vivo *brain hippocampus slice mimic brain structure better than 3D extracellular matrix model. Here we have established an *ex vivo *system, namely rat brain hippocampus slice model, which mimic the in vivo brain structure and microenvironment (Figure 4). This system shows that it can support migration and growth, maintain the survival of stem cells, migrate toward brain tumor cells and drive their differentiation for 30 days (Figure 5), which enable us to study how stem cells target the brain tumor cells. The large dimension with intact fiber track of rat brain hippocampus slice allows detailed mapping the stem cell targeting pathways (Figure 4). This observation can be evaluated *in vivo *animal brain tumor model coupled with a real-time tracking system [43].

## Implication of using organotypic slice platform

Lack of a standardized platform for evaluation of stem cell capacity to target brain tumors may contribute to controversial and inconsistent results. Organotypic slices have the advantage of being easier to use and manipulate than *in vivo *models such as rodents and primates. Moreover, modified organotypic slice systems allow cellular and molecular assessment, which enables, for example, the identification of factors that control neuronal adhesion, acquisition of cell-specific phenotypes, regulation of axonal and dendritic patterning, and development of tumor diseases. Organotypic slices may also be used to study cell migration, cell differentiation, cell-induced tissue injury repair, or even cell susceptibility to drugs. Based upon our preliminary studies, we postulate that a novel stem cell "therapeutic activation state" that is effective for targeting brain tumors can be thoroughly determined by defining associated molecular, biochemical and biological events with an innovative modified organotypic slice system. Establishment of such a standardized platform technology will enable the rapid evaluation of stem cell potential for targeting specific pediatric brain tumors as well as other pathological conditions.

In addition, the proposed platform can be integrated into a work flow to program stem cells to become a clinically relevant stem cell sub-population capable of homing and migration toward tumors (Figure 1). We can determine the migratory parameters by using *in vitro, ex vivo*, and *in vivo *tumor models. Stem cell migration can be critically assessed with physiologically relevant chemokine gradients utilizing microfabricated fluidic chamber systems. *Ex vivo *correlation of chemokine receptors and matrix remodeling capacity can be determined using our innovative organotypic slice microenvironment by which the organ-like microenvironment within the fresh human brain tumor microenvironment can be reconstructed with a real-time monitor. These results can be compared to in vivo *intracranial *brain tumor xenograft models in immunosuppressed mice. The organotypic slice cultures can be used to facilitate the several real-time assessments of cellular, molecular, phenotypic, biochemical, and development characteristics, which enable, for example, the identification of factors that control stem cell adhesion, acquisition of cell-specific phenotypes, and regulation of axonal and dendritic patterning, integration and engraftment of stem cells within tumors. Organotypic slices may also be used to study cell migration, cell differentiation, cell-induced tissue injury repair, or even cell susceptibility to therapeutics.

## Perspectives and future directions

Emerging evidence indicates that stem cells may be a revolutionary therapy for treatment of malignant brain tumors compared with traditional therapies of chemotherapy, radiotherapy and surgery. However, current technologies unfortunately do not consistently deliver the potential clinical benefits of stem cell transplantation for targeting treatment of brain tumors. To begin to unravel the biological behavior of stem cells, we have proposed a conceptual *ex vivo *organotypic slice platform to specifically select stem cell sub-population for targeting brain tumors, which complements the design of traditional *in vivo *animal model studies. Within this experimental framework, data showing the myriad of factors in regulating the complex targeting and migration pattern will shape the traditional biological models. Information obtained will allow us to further explore the detailed mechanisms underlying the perspective roles of stem cell migration for targeting brain tumors. Advances in diagnosis and treatment of childhood cancers are expected to emerge from these coordinated stem cell studies, hopefully culminating in better cancer survival prognosis with a reduction in the risks of acute and late adverse consequences of current treatment.

## Abbreviations

AHSCT: autologous hematopoietic stem cell transplant; ASCT: autologous stem cell transplantation; BBB: blood brain barrier; BNDF: brain-derived neurotrophic factor; CNS: central nerve system; CXCR-4, chemokine (C-X-C motif) receptor 4, a.k.a., SDF-1 receptor; ECM: extra cellular matrix; eNSC: embryonic stem cell-derived human neural stem cells; HDC: high-dose chemotherapy; hNSC: human brain-derived neural stem cells; MMP: matrix metalloproteinase; mNSC: brain-derived mouse neural stem cells; MSC: bone marrow derived mesenchymal stem cells; NGF: nerve growth factor; NSC: neural stem cells; 3D: three dimensional extra-cellular matrix microenvironment; SCF: stem cell factor; SDF-1: stromal derived factor-1 (chemokine), a.k.a., CXCL12.

## Competing interests

The authors declare that they have no competing interests.

## Authors' contributions

SCL conceived of the study, designed, performed, drafted, and revised the entire manuscript, WGL contributed to conception and design, analysis and interpretation of data, as well as helped to draft the manuscript. All authors read and approved the final manuscript.

## Supplementary Material

Additional file 1 Table 2. Current Clinical trials Using Stem Cells for Treatment of Pediatric Brain Tumors.The data provided represent the current status of stem cell therapy of pediatric brain tumors worldwide (The data are from ClinicalTrials.gov, a registry of federally and privately supported clinical trials conducted in the United States and around the world. HSC: Hematopoietic Stem Cell; PBSC: peripheral blood stem cells) Click here for file
